# Accuracy of the GENEActiv Device for Measuring Light Exposure in Sleep and Circadian Research

**DOI:** 10.3390/clockssleep2020012

**Published:** 2020-04-12

**Authors:** Julia E. Stone, Elise M. McGlashan, Elise R. Facer-Childs, Sean W. Cain, Andrew J. K. Phillips

**Affiliations:** Turner Institute for Brain and Mental Health, School of Psychological Sciences, Monash University, Clayton 3800, Victoria, Australia; julia.stone@monash.edu (J.E.S.); elise.mcglashan@monash.edu (E.M.M.); elise.facer-childs@monash.edu (E.R.F.-C.); sean.cain@monash.edu (S.W.C.)

**Keywords:** light, actigraphy, ambulatory monitoring, chronobiology, dosimeter

## Abstract

Light is a variable of key interest in circadian rhythms research, commonly measured using wrist-worn sensors. The GENEActiv Original is a cost-effective and practical option for assessing light in ambulatory settings. With increasing research on health and well-being incorporating sleep and circadian factors, the validity of wearable devices for assessing light environments needs to be evaluated. In this study, we tested the accuracy of the GENEActiv Original devices (*n* = 10) for recording light under a range of ecologically relevant lighting conditions, including LED, fluorescent, infrared, and outdoor lighting. The GENEActiv output had a strong linear relationship with photopic illuminance. However, the devices consistently under-reported photopic illuminance, especially below 100 lux. Accuracy below 100 lux depended on the light source, with lower accuracy and higher variability under fluorescent lighting. The device’s accuracy was also tested using light sources of varying spectral composition, which indicated that the device tends to under-report photopic illuminance for green light sources and over-report for red light sources. Furthermore, measures of photopic illuminance were impacted by infrared light exposure. We conclude that the GENEActiv Original is suitable for mapping light patterns within an individual context, and can reasonably differentiate indoor vs. outdoor lighting, though the accuracy is variable at low light conditions. Given the human circadian system’s high sensitivity to light levels below 100 lux, if using the GENEActiv Original, we recommend also collecting light source data to better understand the impact on the circadian system, especially where participants spend prolonged periods in dim lighting.

## 1. Introduction

Light is the key zeitgeber (time cue) responsible for synchronizing the circadian clock [1] and is therefore a critical measure in studies assessing circadian outcomes. The human circadian system is highly sensitive to light, with its dynamic range overlapping with typical indoor light levels [2,3], and peak sensitivity to blue light [4,5]. Accurate assessment of an individual’s light exposure profile, including under typical indoor lighting, is therefore essential to understanding the impact of light on the circadian system.

In field-based sleep and circadian studies, light exposure is currently widely measured using wrist-worn devices (e.g., [6,7,8,9,10]), due to their convenience and availability. GENEActiv devices are an affordable tool commonly used for this purpose, with 37 studies since 2014 having used the device’s light outputs (Google Scholar search terms “GENEActiv”+“light”+“lux” or “GENEActiv”+“light”+“lx”). An advantage compared to other actigraphic devices is the ability to use raw data with open-access tools developed for analysis and data visualization [11,12,13,14].

Previous studies have characterized the linearity of the GENEActiv light sensor’s response across high intensities [15], or its response to specific narrow-band light sources [16]. However, field studies typically report that participants spend a large amount of time in relatively dim lighting (<100 lux [10]), which is composed primarily of broad-spectrum light sources (e.g., LEDs or fluorescents). We assessed the accuracy of the GENEActiv light sensor using light sources commonly found in home, school, and occupational settings (LED and fluorescent light sources), and in natural daylight, compared against a gold-standard measure. In particular, accuracy was assessed across the circadian system’s dynamic range, including lower illuminances (<100 lux). We further assessed accuracy in light conditions with varying spectral composition: blue (480 nm), green (520 nm), red (635 nm), and infrared.

## 2. Materials and Methods

### 2.1. Devices

Ten GENEActiv Original devices (Activinsights, Cambridgeshire, UK) were tested side by side in a controlled laboratory setting. GENEActivs have a silicon photodiode sensor, with wavelength range 400–1100 nm, reported range of 0–3000 lux, 5-lux resolution, and ±10% accuracy at 1000-lux calibration (manufacturer specifications). All devices were configured on the same computer, and set to record at 30 Hz with the same starting clock time. Data were exported and converted to 15-s epochs using GENEActiv Windows Software from Activinsights. A light meter (J17 Luma Color, Oregon) was used as the gold-standard measurement of photopic illuminance (lux). Spectral measurements were taken using an MK350N spectrometer (UPRTek, Zhunan, Taiwan).

### 2.2. Light Stimuli

Assessments of linearity were taken over a range of broadband illumination conditions, using both fluorescent and LED light sources. The fluorescent light source was a ceiling-mounted casing containing two Philips 4100 K fluorescent bulbs (Master TL5 HE 28W/840 cool lights, Philips Lighting, Amsterdam, Netherlands). Neutral density filters were used to achieve the 10-lux and <3-lux conditions under this light source (209 Neutral Density Filter, LEE, Hampshire, UK). The LED light source was a single square, ceiling-mounted source (CCT: 4289 K, peak wavelength: 451 nm). Maximum illumination conditions were selected based on the highest illuminance that could be achieved using a single light source for fluorescent and LED lights, respectively. A broad range of illuminations below 100 lux were tested due to the high sensitivity of the human circadian system to light, even in low indoor light conditions [2,3]. To assess performance in outdoor daylight, the GENEActivs were tested on an overcast day in September 2019 for two minutes in the afternoon (14:43–14:55 h, Australian Eastern Standard Time, Clayton, Victoria, Australia, Latitude/Longitude -37.900029/145.129918).

To assess performance under extreme spectral conditions, sensors were tested under two circular LED light sources (PhotonStar LED Group, Romsey, UK) set to a constant illuminance of ~120 lux, with varying spectral qualities (peak wavelengths: 470 nm ([blue; FWHM =~28], 520 nm [green; FWHM = ~38], and 630 nm [red; FWHM = ~20]). Light characteristics including effective illuminances are presented in Table 1. While testing, we found that the GENEActiv sensors are sensitive to infrared (IR) light sources. To confirm this observation, we tested GENEActivs in darkness, with and without IR light sources. For the IR test, a Fixed Dome Camera (Vivotek FD8163), which used built-in IR illuminators (effective up to 15 metres; 8 IR LEDs; triggered at ~10 lux), was positioned in the testing room above the devices. 

**Figure 1:**
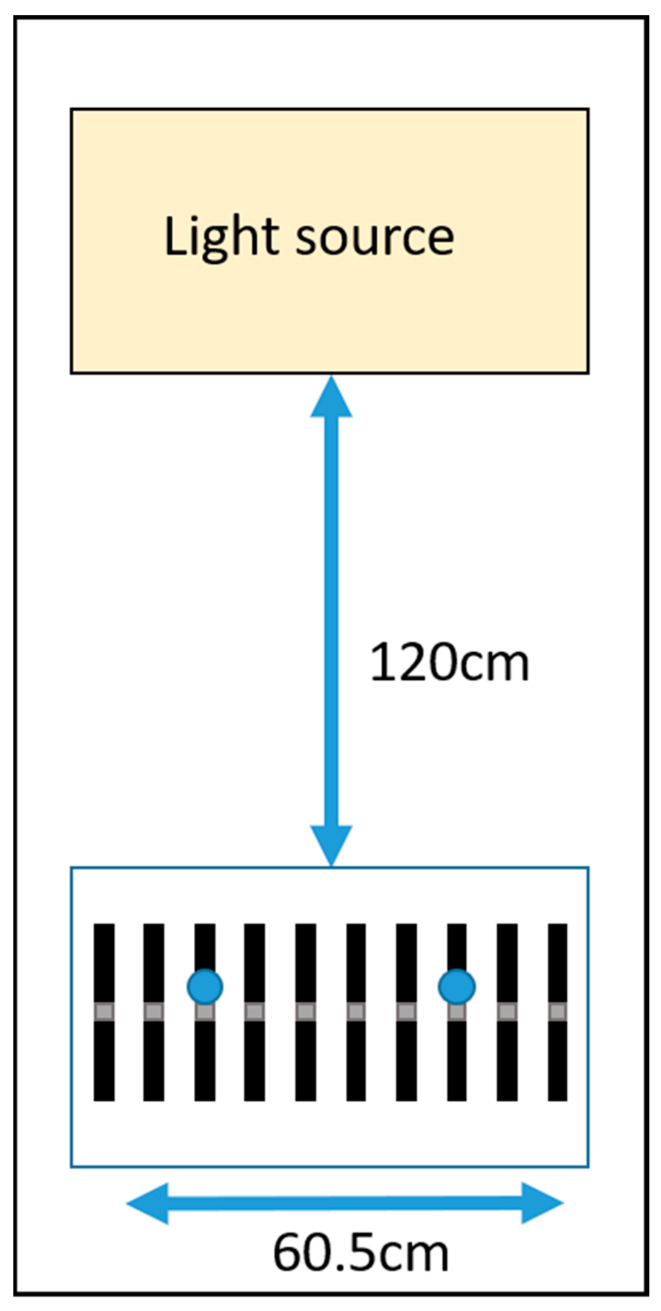
Diagram of device testing configuration. Devices (*n* = 10) were evenly distributed along a white 56 × 60.5 cm board, with sensors facing upwards towards the light source. The board was 120 cm directly beneath the main light source (or centred between light sources in the case of the spectral performance assessment). Lux meter readings were taken at two points along the devices, marked by blue circles. An infrared camera was located on the ceiling next to the light source. Walls surrounding the board and light source were covered in matte black material. The device configuration on the board was identical for the outdoor light test.

Walls were covered in matte black sheets, to minimize indirect illumination from reflective surfaces. Devices were fixed to a white 56 by 60.5 cm board using Velcro, situated directly underneath the light source (Figure 1). Device light sensors were 120 cm directly beneath the light source, with sensor facing up. For the outdoor light test, devices were aligned in the same configuration and placed outdoors on an overcast day. 

### 2.3. Protocol 

Devices were tested under the following light conditions: (i) <3, 10, 30, 50, 100, 200, 400, and 1000 lux, under broad-spectrum fluorescent white light; (ii) <3, 10, 30, 50, 100, 200, and 300 lux under LED white light; (iii) in darkness (0 lux; all lights off); (iv) outdoors in overcast conditions (~21,500 lux); and (v) at ~120 lux at 470 nm (blue), 520 nm (green), and 630 nm (red). Devices were in each light condition for 2 min, to obtain at least 8 independent data points, logged at 15-s intervals. 

To control for variation in light intensity between devices due to angle from light source, lux meter recordings were made at two points along the device line-up (Figure 1). The average value was used for error calculations.

### 2.4. Data Analysis 

Data were exported in 15-s epochs. For each two-minute testing period, the first and last epochs were discarded, and the average of the remaining data points was used for each device. Measurement error for each sensor was calculated by subtracting the device average value from the average lux meter reading. The linear associations between lux meter and GENEActiv lux output were calculated using Pearson’s correlations. The percentage of measured lux (lux meter) reported by the GENEActivs was calculated for each device. Variation between devices was examined by calculating the inter-class correlation coefficient (ICC) as a measure of relative reliability. The standard error of measurement (SEM) and the minimal detectable change (MDC) were calculated as measures of absolute reliability and sensitivity to change [18], respectively. All analyses were conducted using Matlab R2018b (Mathworks, Natick, MA, USA).

## 3. Results

### 3.1. Linearity Test (LED and Fluorescent Light Sources)

**Figure 2:**
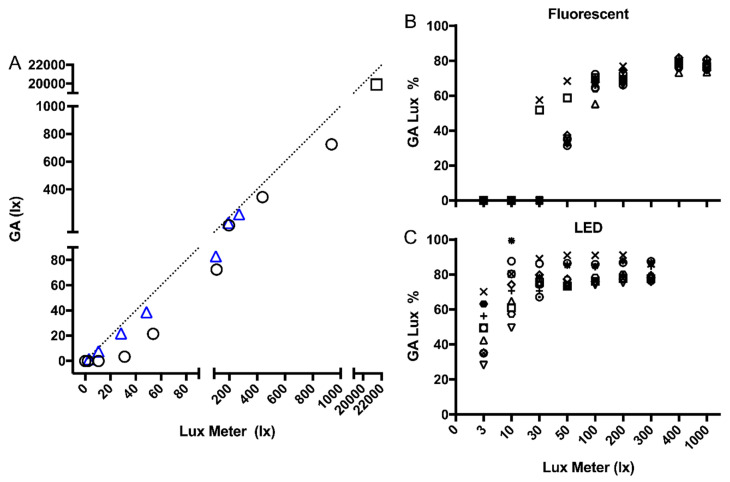
Linearity test of GENEActivs under fluorescent and LED bulbs, and outdoors. (**A**) Mean GENEActiv lux under fluorescent light (circles), LED light (triangles), and outdoor overcast light (square). Dotted line represents perfect agreement. (**B**) GENEActiv lux (*n* = 10) relative (%) to the lux meter under fluorescent light; each symbol represents one device. (**C**) GENEActiv lux (*n* = 10) relative (%) to the lux meter under LED light; each symbol represents one device.

GENEActiv output in lux was compared to the gold-standard measure of photopic lux using a luxometer under the same light conditions. GENEActiv sensors had a strong linear relationship with lux meter readings under both fluorescent (*r* = 0.999, *p* < 0.0001) and LED light sources (*r* = 0.999, *p* < 0.0001). The GENEActivs consistently under-reported light intensity (lux), under both indoor light sources and outdoors (Table 2). This was more pronounced under lower light intensities, particularly under fluorescent lighting (Figure 2). Under the fluorescent light source, all GENEActivs reported 0 lux under the 10 and 3 lux conditions, and eight sensors (80%) reported 0 lux under the 30 lux condition (Figure 2B). This pattern was not observed under the LED light source, which showed higher accuracy at low light levels (Figure 2C). We observed very high reliability between devices under both the fluorescent (ICC 0.998, 95% CI 0.995–0.999) and LED (ICC 0.994, 95% CI 0.985–0.999) light sources. 

### 3.2. Variation in Spectral Composition

**Figure 3:**
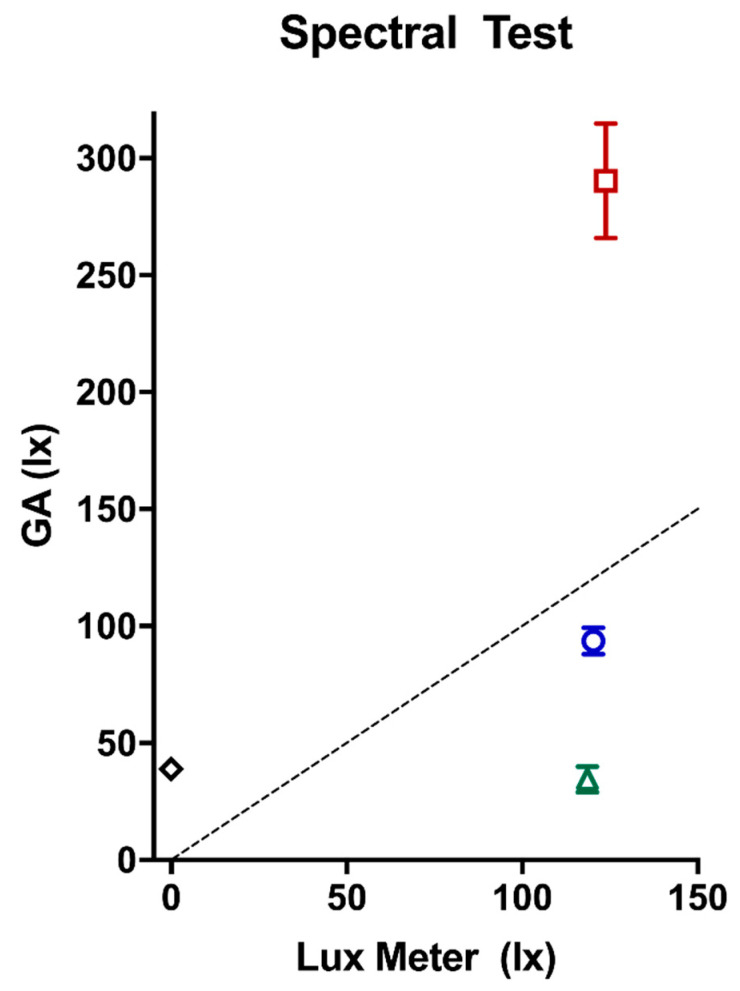
GENEActiv output under ~120 photopic lux with varying wavelength: red (630 nm), blue (470 nm), and green (520 nm), with error bars (standard deviation from the mean). Diamond indicates GENEActiv output under infrared light at 0 lux (note the error bars for infrared are very small and therefore difficult to see; see Table 3). The dotted line indicates perfect agreement between the lux meter and GENEActiv output.

When varying spectral composition (at ~120 lux), we found that GENEActivs over-reported photopic illuminance by 170 ± 24.58 lux under 630 nm (red); and underreported by 26.69 ± 5.66 lux under 470 nm (blue), and by 85.87 ± 5.61 lux under 520 nm (green; see Figure 3). 

In addition, while testing under both the fluorescent and LED light sources, we discovered that the GENEActiv sensors are sensitive to IR light sources, resulting in substantially higher output than measured by the lux meter. Under the 0-lux condition with an IR light source from a wall-mounted camera, GENEActiv devices reported on average 39.15 lux (Table 3). 

## 4. Discussion 

In this report, we tested the accuracy of the now widely used GENEActiv Original for recording light under a range of ecologically relevant lighting conditions. We found that, while the device displays very high inter-device agreement and a strong linear relationship for high light intensities, it tended to under-report photopic illuminance below 100 lux, with the degree of inaccuracy depending on the type of light source. The device’s accuracy was also tested using lights of varying spectral composition (red, green, and blue), which indicated that the device tends to under-report photopic illuminance for green sources and over-report for red. Furthermore, measures of photopic illuminance were heavily impacted by the presence of IR sources. These findings will help to improve interpretation of assessments using this sensor, which is critical in research studies with a circadian component.

The human circadian system responds non-linearly to light intensity, with high sensitivity to light levels below 100 lux [2,3,19]. Consequently, it is important for devices used in sleep and circadian studies to accurately capture light intensities at these levels, which are typically experienced in everyday life. Consistent with a previous study [15], we found that the GENEActiv Original’s output has a strong linear relationship with photopic illuminance at higher light levels (≥200 lux). However, this relationship breaks down at lower light intensities, where its accuracy is highly dependent on the type of light source. While the previous study examined performance of the GENEActiv Original for an LED light source, we compared LED and fluorescent light sources (two sources commonly used in homes, schools, and occupational settings). We found that linearity of the response was maintained for the LED light source down to 10–30 lux, although with higher inter-device variability at 10 lux. For the fluorescent light source, however, linearity of the response was only maintained down to ~100 lux, with higher inter-device variability at 50 lux, 80% of devices reporting zero values at 30 lux; and 100% of devices reporting zero values at 10 lux and below. Future work could expand this assessment to include additional types of light sources, including light-emitting electronic devices. These findings indicate that performance of the device across the circadian system’s dynamic range is highly dependent on the specific type of light source.

Although it is recognized that non-visual responses to light involve a distinct pathway from visual responses to light [17], the use of light-measuring devices that report photopic illuminance remains widespread in circadian research. Light responsiveness of the circadian system is dependent on the photopigment melanopsin [20,21,22]. The GENEActiv Original is not specifically attuned to measuring responses of specific photopigments in the human eye, including melanopsin. A previous investigation of spectral sensitivity across devices demonstrated that most field-based devices, including the GENEActiv Original, do not provide adequate outputs to accurately infer the impact of light on melanopsin [16]. Understanding how the reported photopic illuminance may be biased by the spectral qualities of a light source is important and would help to generalize our findings to other light sources that were not tested here. We compared the photopic illuminance read-outs of the GENEActiv Original for red, green, and blue light sources, finding that the device tended to underestimate photopic illuminance for green light and overestimate for red. Furthermore, measures of photopic illuminance were heavily impacted by the presence of an IR source. The sensitivity of the GENEActiv Original to IR light sources is consistent with a previous report [16], and due to the silicon photodiode’s wide response range (400–1100 nm). This property of the sensor has important implications for use of the device in typical home and workplace settings, where IR motion detectors may be present, with or without knowledge of the participant. In such settings, the device could over-report photopic illuminance in rooms that are dark. 

## 5. Conclusions

In summary, we find that the GENEActiv Original is likely a reliable device for discriminating very bright from very dim light conditions (e.g., indoor vs. outdoor light). However, its accuracy is more variable at lower light levels that commonly occur in the home, particularly with fluorescent light sources. The device is a potentially useful, low-cost alternative to other devices for monitoring patterns of light exposure over time within individuals. Given the human circadian system’s high sensitivity to light levels below 100 lux, however, we advise caution when interpreting light data collected at low indoor light levels. Investigators should also consider collecting information about the types of light sources present when using the device for field studies, especially where participants spend prolonged periods indoors.

## Figures and Tables

**Table 1:** Spectral characteristics, irradiance, and effective illuminance for human photopigments for each of the light sources at ~100 lux.

	Peak (nm)	Photopic Illuminance (lux)	Irradiance (µW/cm^2^)	S Cone	Melanopsin ipRGC	Rod	M Cone	L Cone
Fluorescent	545	100.33	36.30	66.99	59.40	73.04	89.04	94.81
LED	450	100.00	31.48	64.54	72.75	80.06	92.38	97.25
Red	630	111.00	57.31	1.57	1.82	4.11	41.09	141.72
Green	520	114.78	25.76	8.28	119.59	140.84	127.00	97.71
Blue	470	106.56	86.88	464.61	605.06	454.05	253.92	142.74

Note: Spectral measurements taken using an MK350N spectrometer (UPRTek, Zhunan, Taiwan), and effective illuminances/irradiance for each light source, calculated using the Lucas Toolbox [17]. Effective illuminances represent light intensity relative to the spectral sensitivity of each of the human photopigments.

**Table 2:** Summary statistics across *n* = 10 GENEActiv devices under broad-spectrum white fluorescent and LED light sources.

Light Source	Target Condition (lux)	Lux Meter	Mean ± SD Lux	SE Lux	MDC	Mean ± SD Error	Mean ± SD % of Lux Meter
**Dark**							
	0	0.00	0.00 ± 0.00	0.00	0.00	0.00 ± 0.00	100 ± 0.00
**Fluorescent**							
	3	2.10	0.00 ± 0.00	0.00	0.00	−2.10 ± 0.00	0.00 ± 0.00
	10	10.60	0.00 ± 0.00	0.00	0.00	−10.60 ± 0.00	0.00 ± 0.00
	30	31.10	3.09 ± 6.89	2.18	6.04	−27.90 ± 7.18	9.94 ± 22.16
	50	53.80	21.36 ± 6.54	2.07	5.73	−32.60 ± 6.87	39.71 ± 12.15
	100	108.15	72.53 ± 5.07	1.60	4.44	−36.02 ± 5.34	67.06 ± 4.69
	200	197.30	140.40 ± 7.32	2.31	6.41	−59.16 ± 7.46	71.16 ± 3.71
	400	438.75	345.24 ± 12.91	4.08	11.32	−97.24 ± 12.97	78.69 ± 2.94
	1000	935.55	726.82 ± 27.14	8.58	23.79	−218.76 ± 27.37	77.69 ± 2.90
**LED**							
	3	2.84	1.36 ± 0.40	0.13	0.35	−1.45 ± 0.40	48.02 ± 14.02
	10	10.48	7.62 ± 1.49	0.47	1.31	−2.86 ± 1.57	72.69 ± 14.22
	30	28.30	21.87 ± 1.81	0.57	1.58	−6.50 ± 1.89	77.29 ± 6.38
	50	48.46	38.56 ± 2.90	0.92	2.54	−9.28 ± 3.05	79.58 ± 5.99
	100	103.35	82.58 ± 5.44	1.72	4.77	−19.48 ± 5.61	79.91 ±5.26
	200	193.60	158.16 ± 10.16	3.21	8.91	−33.12 ± 10.41	81.70 ± 5.25
	300	270.20	218.62 ± 12.17	3.85	10.66	−51.40 ± 12.53	80.91 ± 4.50
**Outdoor**							
	21 500	21437	19909.70 ± 709.74	224.44	622.12	−1527.30 ± 709.74	92.88 ± 3.31

Note: SD = standard deviation, SE = standard error, MDC = minimal detectable change. Standard deviation is zero in cases where devices uniformly reported a value of zero.

**Table 3:** Summary statistics across *n* = 10 GENEActiv devices under blue, red, green, and infrared light conditions.

	Lux Meter	Mean ± SD Lux	SE Lux	MDC	Mean ± SD Error
Blue	120	93.56 ± 5.66	1.79	4.97	−26.69 ± 5.66
Red	124	290.28 ± 24.58	7.77	21.55	170.03 ± 24.58
Green	119	34.38 ± 5.61	1.77	4.92	−85.87 ± 5.61
Infrared	0	39.15 ± 3.70	1.17	3.24	36.86 ± 3.84

Note: SD = standard deviation, SE = standard error, MDC = minimal detectable change.
